# Characterization of the complete mitochondrial genome of *Stenopsyche angustata* (Trichoptera, Stenopsychidae)

**DOI:** 10.1080/23802359.2020.1797564

**Published:** 2020-08-03

**Authors:** Jing-Cai Huang, Xiao-Xue Wang, Xi-Fa Zhong, Yi-Min Li, Hong-Lin Qin, Yu-Jun Wang, Hong Wang

**Affiliations:** a College of Life Science and Technology, Guangxi University, Nanning, PR China; b Guangxi Key Laboratory of Beibu Gulf Marine Biodiversity Conservation, Ocean College, Beibu Gulf University, Qinzhou, PR China

**Keywords:** Trichoptera, caddisfly, phylogenetic tree, mitochondrial genome

## Abstract

Caddisflies of *Stenopsyche angustata* (Trichoptera, Stenopsychidae) are widely distributed in various freshwater bodies and a few species inhabit marine environments. The mitochondrial genome was sequenced by Illumina high-throughput sequencing, and then the complete mitochondrial genome sequence was obtained through splicing and assembly. The mitochondrial genome sequence size was 15,371 bp, comprising 13 protein-coding genes, 22 tRNA genes, two rRNA genes, and a control region. Two of the protein-coding genes (*COX 2* and *nad 5*) had an incomplete termination codon T. In addition, the start codon of all protein-coding genes was ATN, except for the start codon of the *nad4l* gene which was GTG. The base composition of the mitochondrial genome was 41.64% A, 35.03% T, 7.81% G, and 15.52% C.

*Stenopsyche angustata* is large. The larvae are dark brown in general, with narrow elongated heads and short antennae. The heads have dots or streaks with different sizes and shapes. Ninety-five species of *Stenopsyche* McLachlan are known worldwide, of which 65 species are in China, accounting for 65.3% of the genus (Morse et al. 2014). In recent years, research on *Stenopsyche* McLachlan in China has focused on ecological surveys and the identification of male and female larvae (Tian 1985, 1988; Tian and Zheng 1989); research on its mitochondrial genome has been rare, as has the publication of its complete mitochondrial genome. *Stenopsyche* McLachlan is a genus of Stenopsychidae (Xu et al. 2013), most of which live in clean rivers. Its sensitivity to the water environment means that it can be used as an indicator organism to evaluate the current water quality and also detect any change in water quality (John 1972; Li and Zhou 2001; Hoang and Bae 2007; Chen 2013; Lin et al. 2017). It is of great significance for species identification and ecological protection that the mitochondrial genome of *S. angustata* be analyzed.

The *S. angustata* specimen used in this study was collected from Shiwanda Mountain in Guangxi, China (21.80N,107.89E). The specimen has been deposited in Ocean college marine specimen showroom of Beibu Gulf University (Voucher No. BBGC 00012). This study determined the complete sequence of the complete mitochondrial genome of *S. angustata* using Illumina high-throughput sequencing technology and SPAdes version 3.5.0 software . The size of mitochondrial genome was 15,371 bp, which consisted of 13 protein-coding genes, 22 tRNA genes, two rRNA genes, and a control region.

The size and distribution of the mitochondrial genes were similar to those of other insects. Two of the protein-coding genes (*COX 2* and *nad 5*) had an incomplete termination codon T. In addition, the start codon of all protein-coding genes was ATN, except for the start codon of the *nad4l* gene which was GTG. The base composition of the mitochondrial genome was 41.64% A, 35.03% T, 7.81% G, and 15.52% C.

To better understand the phylogenetic position of *S. angustata*, its complete mitochondrial genome sequence (as determined above) was combined with data from the species construction system to construct a phylogenetic tree. Related target species with a complete mitochondrial or near-complete mitochondrial genome (‘near-complete’ means that the coding gene is complete), were downloaded from the National Center for Biotechnology Information (NCBI). The selected mitochondrial genome protein-coding genes of species used for phylogenetic tree construction were selected separately (Generally, the common coding genes of all species are selected for tree construction.). Using RAxML version 8.1.5 software (https://sco.h-its.org/exelixis/web/software/raxml/index.html), the maximum-likelihood (ML) method was used to build the phylogenetic tree using a bootstrap value of 1000. The results are shown in Figure 1, revealing that *S. angustata* is related to the genus Hydropsychidae, both of which prefer fast-current environments.

**Figure 1.**
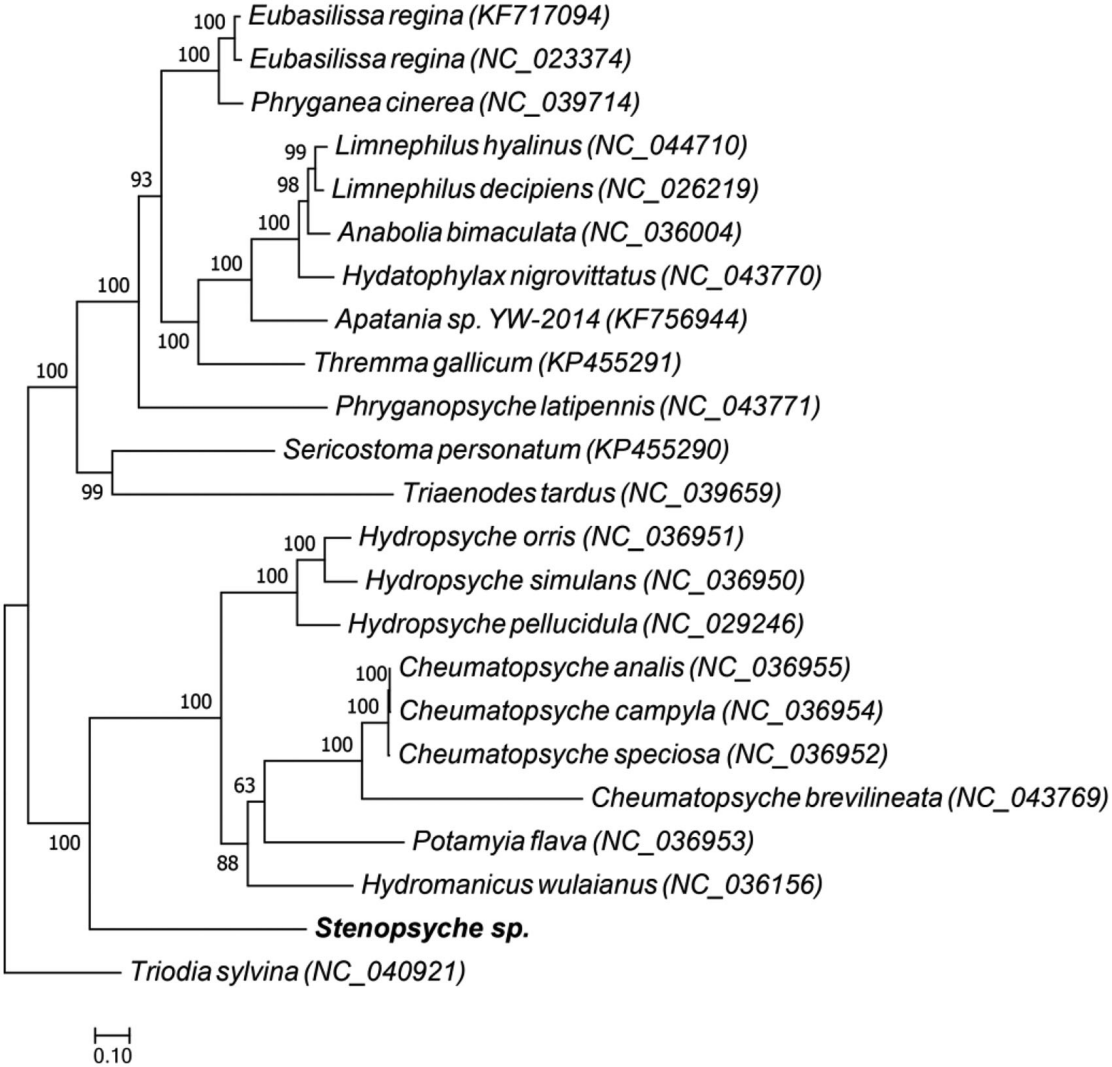
Phylogenetic tree constructed using the maximum-likelihood (ML) method.

## Data Availability

The data that support the findings of this study are openly available in GenBank of NCBI at https://www.ncbi.nlm.nih.gov, reference number MT677866.
